# The First Report of Polymorphisms and Genetic Features of the *prion-like* Protein Gene (*PRND*) in a Prion Disease-Resistant Animal, Dog

**DOI:** 10.3390/ijms20061404

**Published:** 2019-03-20

**Authors:** Sae-Young Won, Yong-Chan Kim, Kiwon Kim, An-Dang Kim, Byung-Hoon Jeong

**Affiliations:** 1 Korea Zoonosis Research Institute, Chonbuk National University, Iksan, Jeonbuk 54531, Korea; gkfh32@jbnu.ac.kr (S.-Y.W.); kych@jbnu.ac.kr (Y.-C.K.); 2 Department of Bioactive Material Sciences, Chonbuk National University, Jeonju, Jeonbuk 54896, Korea; 3 Haemalken Animal Hospital, Yangju, Gyeonggi 11492, Korea; kkw0075@hanmail.net; 4 Cool-Pet Animal Hospital, Anyang, Gyeonggi 14066, Korea; kad7582@hanmail.net

**Keywords:** dog, *prion-like* protein gene, *PRND*, Doppel, linkage disequilibrium, prion

## Abstract

Prion disease has displayed large infection host ranges among several species; however, dogs have not been reported to be infected and are considered prion disease-resistant animals. Case-controlled studies in several species, including humans and cattle, indicated a potent association of prion protein gene (*PRNP*) polymorphisms in the progression of prion disease. Thus, because of the proximal location and similar structure of the *PRNP* gene among the prion gene family, the *prion-like* protein gene (*PRND*) was noted as a novel candidate gene that contributes to prion disease susceptibility. Several case-controlled studies have confirmed the relationship of the *PRND* gene with prion disease vulnerability, and strong genetic linkage disequilibrium blocks were identified in prion-susceptible species between the *PRNP* and *PRND* genes. However, to date, polymorphisms of the dog *PRND* gene have not been reported, and the genetic linkage between the *PRNP* and *PRND* genes has not been examined thus far. Here, we first investigated dog *PRND* polymorphisms in 207 dog DNA samples using direct DNA sequencing. A total of four novel single nucleotide polymorphisms (SNPs), including one nonsynonymous SNP (c.149G>A, R50H), were identified in this study. We also found two major haplotypes among the four novel SNPs. In addition, we compared the genotype and allele frequencies of the c.149G>A (R50H) SNP and found significantly different distributions among eight dog breeds. Furthermore, we annotated the c.149G>A (R50H) SNP of the dog *PRND* gene using *in silico* tools, PolyPhen-2, PROVEAN, and PANTHER. Finally, we examined linkage disequilibrium between the *PRNP* and *PRND* genes in dogs. Interestingly, we did not find a strong genetic linkage between these two genes. To the best of our knowledge, this was the first genetic study of the *PRND* gene in a prion disease-resistant animal, a dog.

## 1. Introduction

Prion diseases, neurodegenerative disorders in humans and animals, are also known as transmissible spongiform encephalopathies (TSEs) characterized by a structural folding change from a normal prion protein (PrP^C^) to a toxic form of prion protein (PrP^Sc^) causing brain lesions [1,2,3]. The most represented TSEs are scrapie in sheep and goats; chronic wasting disease (CWD) in elk and deer [4,5,6,7]; bovine spongiform encephalopathy (BSE) in cattle [8,9]; transmissible mink encephalopathy (TME) in minks [10]; feline spongiform encephalopathy (FSE) in cheetahs, pumas and cats [11,12,13]; and Creutzfeldt-Jakob disease (CJD), fatal familial insomnia (FFI) and Gerstmann-Sträussler Scheinker syndrome (GSS) in humans [14,15]. Interestingly, although dogs and cats have similar prey and habitats, prion disease in dogs has not been reported thus far. Therefore, many studies have been performed in dogs to identify factors that affect the resistance to prion disease.

According to previous studies, susceptibility to prion disease can be influenced by three major factors. One is the amount of prion protein expression. The 12-bp and 23-bp insertion/deletion (indel) polymorphisms in the promoter region of the bovine *PRNP* gene have been strongly associated with the expression level of bovine prion protein and the susceptibility to prion disease in cattle. Overexpression of prion protein based on the haplotype of 23-bp and 12-bp polymorphisms has been considered a vulnerability factor for BSE [8,16,17]. In addition, ablation of prion protein in knockout animals did not cause infection with prion disease [18]. However, there was no significant difference in the expression level of prion protein in the brain between dogs and other animals [19]. Another factor is the genetic characteristic of the prion protein. Polymorphisms of the prion protein gene (*PRNP*) in several species have been shown to be strongly correlated with prion disease susceptibility. In sheep, the haplotypes of codons 136, 154 and 171 affect susceptibility (VRQ, ARQ) or resistance (ARR) to scrapie [20,21]. The goat prion protein codon M142 extends the scrapie incubation period. In addition, codons S146N, R154H and Q222K protect against scrapie [22,23,24,25,26]. In humans, the *PRNP* genotypes of codons 129 and 219 are well known for being factors of CJD susceptibility [27,28]. In prion-resistant species, the dog prion protein D163 residue strongly contributes to prion disease resistance [29,30,31,32,33,34,35]. However, because a high dose of PrP^Sc^ infection converted dog PrP^C^ to PrP^Sc^, it is not sufficient to fully explain prion disease resistance in dogs [19]. The other factor is other candidate genes besides prion protein that can affect the susceptibility to prion diseases. Among them, the *prion-like* protein gene (*PRND*) is a potent candidate gene that may play a role in prion disease susceptibility. According to case-controlled studies comparing the genetic distribution of *PRND* gene polymorphisms in codons 26, 56, 132 and 174 and 3’ untranslated region (UTR) +28, these polymorphisms were involved in the susceptibility to scrapie, BSE and sporadic CJD in ruminants and humans [36,37,38,39,40]. In addition, recent studies have reported a strong linkage disequilibrium (LD) between the *PRNP* gene and *PRND* gene in prion disease-susceptible species, sheep and goats [38,41]. However, no study has been conducted on the genetic characteristics of *PRND* in dogs. Thus, the investigation of the genetic characteristics of the *PRND* gene in dogs as a prion disease-resistant species will be a very important baseline study to obtain clues on the progression of prion disease.

In the present study, we investigated the dog *PRND* genotype, allele and haplotype frequencies of single nucleotide polymorphisms (SNPs). We also annotated nonsynonymous SNPs using in silico analysis tools, PolyPhen-2 [42], PROVEAN [43,44] and PANTHER [45]. In addition, we performed LD tests among *PRND* SNPs and analyzed major haplotypes of *PRND* SNPs. Furthermore, we measured the LD value between the *PRNP* gene and *PRND* gene in dogs.

## 2. Results

### 2.1. Investigation of Genetic Characteristics of the PRND Gene in the 207Dogs

**Figure 1:**
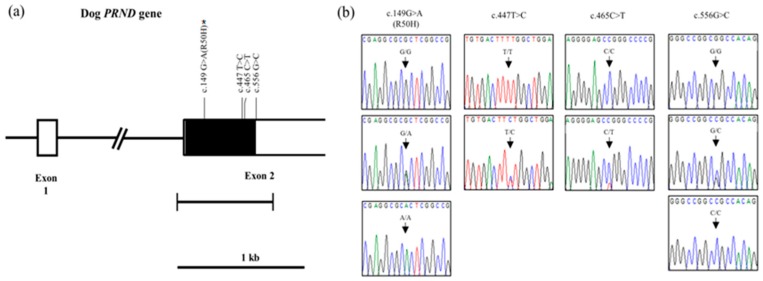
Gene map and polymorphisms identified in the dog *prion-like* protein gene (*PRND*) on chromosome 24. (**a**) The open reading frame (ORF) is indicated by a shaded block, and the 5′ and 3′ untranslated regions (UTRs) are indicated by white blocks. Horizontal bars with edges indicate the regions sequenced. Arrows indicate the novel polymorphisms found in this study. The asterisk indicates the nonsynonymous single nucleotide polymorphism (SNP) of the dog *PRND* gene. (**b**) Electropherogram of four novel SNPs: c.149G>A (R50H), c.447T>C (F149F), c.465C>T (A155A) and c.556G>C, identified in this study. Four colors indicate individual bases of DNA sequence using an ABI 3730 automatic sequencer (blue: cytosine, red: thymine, black: guanine, green: adenine).

The dog *PRND* gene is composed of two exons. To investigate polymorphisms of the *PRND* gene in dogs, we performed direct sequencing analysis targeting exon 2, which contains the full length ORF. We found a total of four novel SNPs: c.149G>A, c.447T>C, c.465C>T in the ORF and c.556G>C in the 3′ UTR of the *PRND* gene (Figure 1a,b).

Among the four SNPs, c.149G>A (R50H) is a nonsynonymous SNP. Detailed values of the genotype and allele frequencies of the dog *PRND* gene are described in Table 1.

We also investigated LD among the four dog *PRND* SNPs using (|D’|) and r^2^ values. All four SNPs have strong LDs with D’ values of 1.0 according to the (|D’|) value. In addition, the r^2^ value showed a value of 1.0 between c.149G>A and c.556G>C and between c.447T>C and c.465C>T (Table 2). Next, we performed haplotype analysis of the dog *PRND* gene. The two major haplotypes were identified, including GGTC and ACTC, and the GGTC haplotype had the highest frequency (87.4%) in the dog *PRND* gene (Table 3).

**Figure 2:**
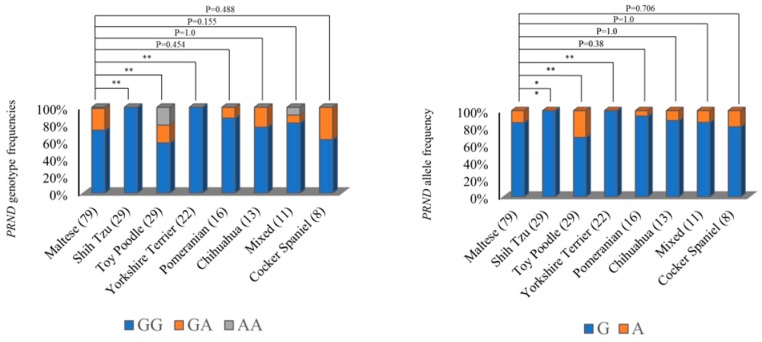
Comparisons of genotype and allele frequencies of c.149G>A (R50H) among eight dog breeds. Differences in the c.149G>A (R50H) genotype and allele frequencies among eight dog breeds (Maltese, Shih Tzu, Toy Poodle, Yorkshire Terrier, Pomeranian, Chihuahua, Cocker Spaniel, and Mixed dogs) were calculated by the chi-square test using Statistical Analysis Software (SAS) version 9.4. Parentheses indicate the number of dogs. Statistically significant differences are indicated below. * *p* value < 0.05, ** *p* value < 0.01, *** *p* value < 0.001.

Next, we compared the genotype and allele frequencies of the c.149G>A (R50H) SNP in the dog *PRND* gene among eight dog breeds using the chi-square test. Maltese has no significant difference from Pomeranian (*p* = 0.454), Chihuahua (*p* = 1.0), Mixed (*p* = 0.155), and Cocker Spaniel (*p* = 0.488) in genotype frequency. However, Maltese has a significantly different genotype distribution with Shih Tzu (*p* = 0.0019), Toy Poodle (*p* = 0.0036) and Yorkshire Terrier (*p* = 0.0067). In addition, the allele frequency of Maltese was significantly different from that of Shih Tzu (*p* = 0.0014), Toy Poodle (*p* = 0.0058) and Yorkshire Terriers (*p* = 0.005) (Figure 2).

### 2.2. Analysis of the Genetic Linkage between SNPs of the PRNP and PRND Genes

**Figure 3:**
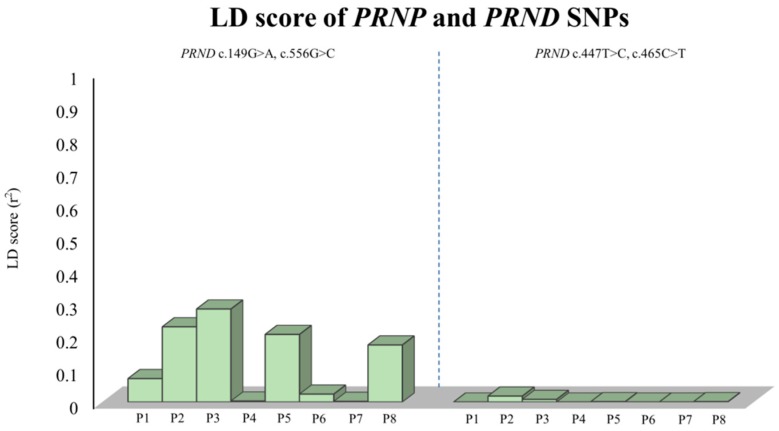
The linkage disequilibrium (LD) scores between polymorphisms of the *PRND* gene and those of the *PRNP* gene. LD scores with r^2^ values between *PRND* and *PRNP* polymorphisms in dogs. P1–P8 indicate *PRNP* polymorphisms as follows: P1, c.190in/del (codon 64); P2, c.198T>C (codon 66); P3, c.301A>G (codon 101); P4, c.372G>A (codon 124); P5, c.489C>G (codon 163); P6, c.545A>G (codon 182); P7, c.546C>A (codon 182); and P8, c.729T>C (codon 243).

To examine whether dog *PRND* SNPs have a genetic linkage with SNPs of the dog *PRNP* gene, we carried out LD analysis between SNPs of these two genes. Figure 3 summarizes LD analysis using the r^2^ value. Interestingly, all *PRND* SNPs showed weak LD with *PRNP* SNPs (r^2^ value: below 0.3). Detailed values are described in Supplementary Tables S1 and S2.

### 2.3. Measurement of Protein Functional Alterations Induced by Nonsynonymous SNPs

We measured the damage of nonsynonymous SNPs (R50H) using PolyPhen-2, PROVEAN and PANTHER. PolyPhen-2 predicted R50H to be ‘benign’ and scored 0.051. The PROVEAN program analyzed R50H to be ‘neutral’, with a score of -1.065. PANTHER predicted R50H to be ‘probably benign’ and scored 30 (Table 4).

### 2.4. The Sequence Alignments of Doppel Protein among Several Species

**Figure 4:**
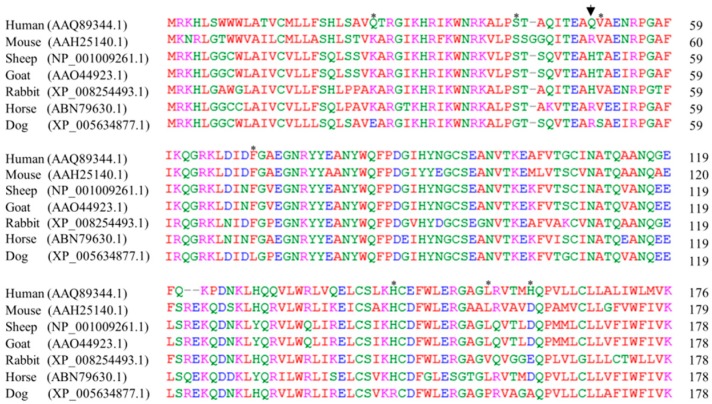
Comparisons of amino acid sequences of *prion-like* protein (Doppel) in human, mouse, sheep, goat, rabbit, horse, and dog. *Prion-like* protein sequences were obtained from GenBank at the National Center for Biotechnology Information (NCBI), including those of human (*Homo sapiens*, AAQ89344.1), mouse (*Mus musculus*, AAH25140.1), sheep (*Ovis aries*, NP_001009261.1), goat (*Capra hircus*, AAO44923.1), rabbit (*Oryctolagus cuniculus*, XP_008254493.1), horse (*Equus caballus*, ABN79630.1), and dog (*Canis lupus familiaris*, XP_005634877.1). Doppel protein sequences were aligned among various species using ClustalW2. Colors symbolize the chemical properties of amino acids (blue: acidic; red: small and hydrophobic; magenta: basic; green: hydroxyl, sulfhydryl, amine and glycine). The arrow denotes the nonsynonymous single nucleotide polymorphism (SNP) (c.149G>A, R50H) found in this study. Asterisks indicate dog-specific residues.

Finally, we performed amino acid sequence alignment of prion-like protein (Doppel) between dogs and other species (human, mouse, sheep, goat, rabbit, and horse). When compared to other species, dog *prion-like* protein has eight dog-specific amino acids, including leucine (L) in codon 18, glutamic acid (E) in codon 25, glycine (G) in codon 42, serine (S) in codon 51, leucine (L) in codon 70, arginine (R) in codon 146, proline (P) in codon 157, and alanine (A) in codon 162 (Figure 4).

## 3. Discussion

The *PRND* gene is in the same family of genes as the *PRNP* gene. Structural similarities with the *PRNP* gene and an association with prion disease have been reported in previous studies [44]. Recently, a strong genetic linkage between the *PRNP* gene and *PRND* gene was identified, and scrapie-associated SNPs were strictly linked to the genotype of the *PRND* gene [38,41]. Because those studies have been performed in prion disease-susceptible species, it is elusive whether strong genetic linkage was a prion disease-susceptible factor or a general property among prion gene families. Here, we first investigated dog *PRND* gene polymorphisms and analyzed the genetic linkage between the dog *PRNP* gene and the dog *PRND* gene. We found a total of four novel SNPs, including one nonsynonymous SNP. Among the four SNPs, the R50H SNP has already been identified in cattle. In addition, four *PRND* SNPs have a strong genetic linkage and construct two major haplotypes (Table 2 and Table 3). Interestingly, genotype and allele frequencies are significantly different among eight dog breeds (Figure 2). Next, we investigated LD analysis between the *PRNP* gene and *PRND* gene. Notably, there was no strong LD between *PRND* and *PRNP* SNPs using r^2^ analysis. Previous studies have indicated that prion disease-susceptible species, such as sheep and goats, have a strong LD between the *PRNP* gene and the *PRND* gene, which is very interesting. Furthermore, because dogs have a relatively short genetic distance between the *PRNP* gene and *PRND* gene compared to those of other species (cattle: 26 kb; goat: 21 kb; sheep: 25 kb; human: 20 kb; horse: 16 kb; dog: 17 kb), weak LD between the *PRNP* and *PRND* genes was assumed not to be induced by the genetic distance between the *PRNP* gene and *PRND* gene. In addition, it is notable that prion disease-resistant animals, including horses and dogs, have a relatively short genetic distance between the *PRNP* gene and *PRND* gene compared to prion disease-susceptible animals. Because only three studies have performed (including the present study) LD analysis between the *PRNP* and *PRND* gene, further investigation of LD in a wide range of animals in the future is highly desirable. The important thing is that dogs did not have strong LD between the *PRNP* gene and the *PRND* gene, unlike prion disease-susceptible species.

Finally, we estimated the nonsynonymous SNP R50H using PolyPhen-2, PROVEAN and PANTHER. Three *in-silico* estimation programs did not predict R50H to be deleterious to doppel protein function. However, previous studies indicated that synonymous SNPs located in codon 26 of the *PRND* gene can impact Doppel function, especially the reproductive ability of sperm [46,47]. In addition, synonymous SNPs may impact transcription efficiency and can affect the phenotype of a protein [48]. Thus, in vivo or in vitro confirmation studies based on genotype, allele and haplotype distributions of the dog *PRND* gene will be needed in the future. In recent studies, the prion gene family member *prion-related* protein gene (*PRNT*), which is located downstream of the *PRND* gene, was expressed in reproductive organs in a manner similar to the *PRND* gene, and a strong genetic linkage with the *PRND* gene has also been reported [49,50,51,52]. Because a strong genetic LD block has been detected in sheep and goats, further confirmation of the genetic linkage between these adjacent genes is also needed in the future.

## 4. Materials and Methods

### 4.1. Ethical Statement

Whole blood samples of 207 dogs, including eight dog breeds (79 Maltese, 29 Shih Tzu, 29 Toy Poodle, 22 Yorkshire Terriers, 16 Pomeranian, 13 Chihuahua, 11 Mixed, 8 Cocker Spaniel), were provided by the Anyang cool pet animal hospital in the Republic of Korea. All experimental procedures were accredited by the Institute of Animal Care and Use Committee of Chonbuk National University (CBNU 2018-062) and approved on 27 July 2018.

### 4.2. Genetic Analysis

Genomic DNA was extracted from 200 µL of a whole blood sample using a Hi Yield Genomic DNA Mini Kit (Real Biotech Corporation, Taipei, Taiwan) and a Bead Genomic DNA Prep Kit (Biofact, Daejeon, Korea). Polymerase chain reaction (PCR) was performed with forward primer: 5′-AGAAAGTAACTGCCCCGAGC-3′ and reverse primer: 5′ TTTGGTACCTTGGGGACACG-3′. These primers were designed based on the *PRND* gene sequence from GenBank (Gene ID: 485782) and amplified DNA sequences containing the entire open reading frame (ORF) of the dog *PRND* gene. The length of the PCR products was 688 bp. A 25 µL reaction mixture containing 2.5 µL of 10X Taq DNA polymerase, 1 µL of genomic DNA, 10 pmol each primer, 0.5 µL of a 0.2 µM dNTP mixture, 0.2 µL of *Taq* DNA polymerase, and sterile deionized water, was used. The PCR conditions were as follows: denaturing at 95 °C for 2 min, followed by 34 cycles of 95 °C for 20 s, 63 °C for 30 s, and 72 °C for 1 min 30 s and one cycle of 72 °C for 5 min. The PCR products were separated on a 1% agarose gel stained with ethidium bromide (EtBr), and the PCR products were purified using a FavorPrep GEL/PCR Purification Mini Kit (FAVORGEN, Pingtung County, Taiwan). Purified PCR products were directly sequenced using an ABI 3730 sequencer (ABI, Foster City, CA, USA); sequencing results were visualized using Finch TV software (Geospiza Inc., Seattle, WA, USA), and genotyping was performed.

### 4.3. Statistical Analysis

Genotype and allele frequencies of the dog *PRND* gene were compared among eight dog breeds by chi-square test using SAS 9.4 software (SAS Institute Inc., Cary, NC, USA). Haplotype analysis and linkage disequilibrium calculated by Lewon-tin’s D’ (|D’|) and pairwise linkage disequilibrium (r^2^) were performed using Haploview version 4.2 (Broad Institute, Cambridge, MA, USA).

### 4.4. Analysis of the Genetic Linkage between SNPs of the PRNP and PRND Genes

LD analysis was performed between *PRNP* and *PRND* SNPs. LD scores of the *PRNP* and *PRND* genes were calculated in 174 animals. Next, the genotype distributions of *PRND* were compared with those of the *PRNP* gene, and the difference of distribution was calculated using the chi-square test. All statistical analyses were calculated by Statistical Analysis Software (SAS), version 9.4 (SAS Institute Inc., Cary, NC, USA), and statistically significant differences were determined by *p* value < 0.05.

### 4.5. The Sequence Alignments of Doppel Protein among Several Species

The alignments of the *prion-like* protein (Doppel) sequence were performed by ClustalW2 (http://www.ebi.ac.uk/Tools/msa/clustalo/). The analysis was performed for Doppel protein sequences of human (*Homo sapiens*, AAQ89344.1), mouse (*Mus musculus*, NC_000068.7), sheep (*Ovis aries*, NP_001009261.1), goat (*Capra hircus*, AAO44923.1), rabbit (*Oryctolagus cuniculus*, XP_008254493.1), horse (*Equus caballus*, ABN79630.1), and dog (*Canis lupus familiaris*, XP_005634877.1). All information of these sequences was obtained from GenBank at the National Center for Biotechnology Information (NCBI).

### 4.6. Measurement of Protein Functional Alterations Induced by Nonsynonymous SNPs

PolyPhen-2 (http://genetics.bwh.harvard.edu/pph2/index.shtml), PROVEAN (http://provean.jcvi.org/seq_submit.php), and PANTHER (http://www.pantherdb.org/) programs have evaluated protein substitution by nonsynonymous SNPs. PolyPhen-2 evaluates the effect of amino acid changes according to the position-specific, independent count (PSIC) score and indicates three types of functional changes: “probably damaging”, “possibly damaging” and “benign”. PROVEAN estimates the impact score of nonsynonymous SNPs on protein function, with a score below −2.5 being “deleterious” and a score above −2.5 being “neutral”. PANTHER uses a hidden Markov model (HMM)-based statistical modeling method to measure scores due to amino acid changes. The PANTHER score below −3 indicates “deleterious” and above -3 indicates “neutral”.

## 5. Conclusions

In summary, we investigated SNPs of the dog *PRND* gene. We identified four novel SNPs, including 1 nonsynonymous SNP. A total of 4 SNPs constitute two major haplotypes with strong LD. In addition, we found significantly different distributions of genotype and allele frequencies among eight dog breeds. Furthermore, we evaluated the LD value between the *PRNP* gene and the *PRND* gene in dogs and found relatively weak LD compared to that in prion disease-susceptible animals, such as sheep and goats. Lastly, we performed in silico estimation of the nonsynonymous SNP of the *PRND* gene R50H using PolyPhen-2, PROVEAN and PANTHER. To the best of our knowledge, this is the first genetic study of the dog *PRND* gene.

## Figures and Tables

**Table 1:** Genotype and allele frequencies of *PRND* polymorphisms in dogs.

Polymorphisms	Genotype Frequency, *n* (%)			Allele Frequency, *n* (%)	
c.149G>A (R50H)	GG 164 (79.2)	GA 35 (16.9)	AA 8 (3.9)	G 363 (87.7)	A 51 (12.3)
c.447T>C (F149F)	TT 206 (99.5)	TC 1 (0.5)	CC 0 (0)	T 413 (99.8)	C 1 (0.2)
c.465C>T (A155A)	CC 206 (99.5)	CT 1 (0.5)	TT 0 (0)	C 413 (99.8)	T 1 (0.2)
c.556G>C	GG 164 (79.2)	GC 35 (16.9)	CC 8 (3.9)	G 363 (87.7)	C 51 (12.3)

**Table 2:** Linkage Disequilibrium (LD) among four polymorphisms of *PRND* gene in dogs.

	|D’|			
r^2^	c.149G>A	c.447T>C	c.465C>T	c.556G>C
c.149G>A	-	1.0	1.0	1.0
c.447T>C	0	-	1.0	1.0
c.465C>T	0	1.0	-	1.0
c.556G>C	1.0	0	0	-

**Table 3:** Haplotype frequency of four *PRND* polymorphisms in dogs.

Haplotype	Dogs (*n* = 414)
GGTC	362 (0.874)
ACTC	51 (0.123)
Others	1 (0.003)

**Table 4:** Prediction of non-synonymous polymorphisms in dogs by PolyPhen-2, PROVEAN and PANTHER.

Variation	Method	Score	Prediction
c.149G>A (R50H)	PolyPhen-2	0.051	Benign
	PROVEAN	−1.065	Neutral
	PANTHER	30	Probably benign

## Supplementary Materials

Supplementary materials can be found at https://www.mdpi.com/1422-0067/20/6/1404/s1. Table S1: Linkage Disequilibrium (LD) between polymorphisms (SNPs) of *PRNP* and *PRND* gene with r^2^ value in dogs. Table S2: Linkage Disequilibrium (LD) between polymorphisms of *PRNP* and *PRND* gene with D’ value in dogs.

## Abbreviations

BSE	Bovine spongiform encephalopathy
CJD	Creutzfeldt-Jakob disease
CWD	Chronic wasting disease
FFI	Fatal familial insomnia
FSE	Feline spongiform encephalopathy
GSS	Gerstmann-Sträussler Scheinker syndrome
LD	Linkage disequilibrium
PRND	*Prion-like* protein gene
PRNP	Prion protein gene
SNPs	Single nucleotide polymorphisms (SNPs)
TME	Transmissible mink encephalopathy
TSEs	Transmissible spongiform encephalopathies
