# Supplementary material for: The First Report of Polymorphisms and Genetic Features of the prion-like Protein Gene (PRND) in a Prion Disease-Resistant Animal, Dog
Source: Int J Mol Sci. 2019 Mar 20;20(6):1404. doi: 10.3390/ijms20061404 (PMC6470729; doi:10.3390/ijms20061404)
Supplement: Supplementary file 1 [file ijms-20-01404-s001.zip › ijms-460402-SI.pdf]

**Supplementary Table 1.** Linkage Disequilibrium (LD) between polymorphisms (SNPs) of *PRNP* and *PRND* gene with $r^2$ value in dogs.

| *PRNP* | *PRND* | | | |
|---|---|---|---|---|
| | c.149G>A | c.447T>C | c.465C>T | c.556G>C |
| c.190in/del (codon 64) | 0.071 | 0.0 | 0.0 | 0.071 |
| c.198T>C (codon 66) | 0.228 | 0.018 | 0.018 | 0.228 |
| c.301A>G (codon 101) | 0.282 | 0.008 | 0.008 | 0.282 |
| c.372G>A (codon 124) | 0.003 | 0.0 | 0.0 | 0.003 |
| c.489C>G (codon 163) | 0.205 | 0.001 | 0.001 | 0.205 |
| c.545A>G (codon 182) | 0.024 | 0.0 | 0.0 | 0.024 |
| c.546C>A (codon 182) | 0.002 | 0.0 | 0.0 | 0.002 |
| c.729T>C (codon 243) | 0.173 | 0.001 | 0.001 | 0.173 |

**Supplementary Table 2.** Linkage Disequilibrium (LD) between polymorphisms of *PRNP* and *PRND* gene with D' value in dogs.

| | *PRND* | | | |
|---|---|---|---|---|
| *PRNP* | c.149G>A | c.447T>C | c.465C>T | c.556G>C |
| c.190in/del (codon 64) | 1.0 | 1.0 | 1.0 | 1.0 |
| c.198T>C (codon 66) | 0.552 | 1.0 | 1.0 | 0.552 |
| c.301A>G (codon 101) | 0.916 | 1.0 | 1.0 | 0.916 |
| c.372G>A (codon 124) | 0.999 | 1.0 | 1.0 | 0.999 |
| c.489C>G (codon 163) | 0.695 | 1.0 | 1.0 | 0.695 |
| c.545A>G (codon 182) | 0.67 | 1.0 | 1.0 | 0.67 |
| c.546C>A (codon 182) | 1.0 | 1.0 | 1.0 | 1.0 |
| c.729T>C (codon 243) | 0.681 | 1.0 | 1.0 | 0.681 |
